# An improved technology for monitoring groundwater flow velocity and direction in fractured rock system based on colloidal particles motion

**DOI:** 10.1038/s41598-024-58235-z

**Published:** 2024-04-01

**Authors:** Fei Hu, Chang-Sheng Huang, Ji-Hong Han, Wei Huang, Xuan Li, Bao-Quan Hou, Waseem Akram, Long Li, Xue-Hao Liu, Wei Chen, Zi-Liang Zhao, Jia Zhan, Lian-Shan Xu, Hua Shan, Xiao-Zhe Li, Wen-Jing Han, Zhi-Bin Yin, Zhong-Zhong Wang, Tang-Fu Xiao

**Affiliations:** 1 School of Environmental Science and Engineering, Guangzhou University, Guangzhou, 510006 China; 2 Wuhan Center, China Geological Survey, Wuhan, 430205 China; 3 Central South China Innovation Center for Geosciences, Wuhan, 430205 China; 4 Guangdong Geological Survey Institute, Guangzhou, 510110 China; 5 The Institute of Geological Survey of China University of Geosciences (Wuhan), Wuhan, 430074 China; 6 Tianjin Municipal Engineering Design & Research Institute, Tianjin, 300000 China; 7 Fourth Geological Team of Hubei Geological Bureau, Xianning, 437100 China; 8 The Institute of Hydrogeologic and Engineering Geological of Wuhan, Hubei Province Geological Survey, Wuhan, 430051 China

**Keywords:** Groundwater flow velocities and directions, Fissure water, Hydrogeology, Monitoring and calculation methods, Limit equation method, Hydrology, Hydrology

## Abstract

The colloidal borescope, using colloidal particle motion, is used to monitor the flow velocities and directions of groundwater. It integrates advanced techniques such as microscopy, high-speed photography, and big data computing and enjoys high sensitivity at the micron level. However, In the same well, the groundwater flow velocity monitored by colloidal hole mirror is varies greatly from that obtained by conventional hydrogeological monitoring, such as pumping test. In order to solve this problem, the stability catcher and stratified packer are designed to control the interference of the vertical flow in drilling, and to monitor the flow velocity and direction of groundwater velocity at the target aquifer and target fracture. Five wells with different aquifers and different groundwater types were selected for monitoring in south-central China. The instantaneous velocity and direction are converted into east–west component and north–south component, the average velocity and direction is calculated according to the time of 10 min, and the particle trajectory diagram is established. Based on these results, it proposed a concept of cumulative flow velocity. Using curve-fitting equations, the limits of cumulative flow velocities as the monitoring time tends to infinity were then calculated as the actual flow velocities of the groundwater. The permeability coefficient of aquifer is calculated by using the fissure ratio of aquifer, hydraulic slope and flow velocity, and compared with the permeability coefficient obtained by pumping test. The results are as follows: (1) The variation coefficient of the instantaneous flow velocity measured at the same depth in the same well at different times is greater than that of the time average flow velocity and greater than that of the cumulative flow velocity. The variation coefficient of the actual velocity is the smallest, indicating that the risk of using the actual flow velocity is lower. (2) The variation coefficient of the flow rate monitored at different depths in the same well is mainly controlled by the properties of the aquifer. The more uniform water storage space in the aquifer, the smaller the variation coefficient. (3) The comparison between the permeability coefficient obtained by monitoring and the permeability coefficient obtained by pumping test shows that the flow of structural fissure water controlled by planar fissure is more surface flow, and the results are consistent. When the groundwater flow is controlled by pores and solution gaps, the flow channel is complicated, which is easy to produce turbulent flow, and the result consistency is poor. (4) According to different research accuracy requirements, different monitoring and calculation methods can be selected for different aquifers and groundwater types. Researches show that, the permeability coefficient calculated for the actual flow velocity in well DR01 is the same as that calculated for the pumping test. The aquifer characteristics reflected by the coefficient of variation of the actual flow velocity in the same aquifer are more realistic. The pumping test method obtains the comprehensive parameters of a certain aquifer, and this method can be used to monitor a certain fissure. In this paper, the new technology developed for monitoring, and the new algorithm established for data processing, can accurately obtain the flow velocity and direction of groundwater, using capsule hole mirror monitoring method. The key parameters of hydrogeology can be obtained by using one well, which can reduce the time and cost input and improve the work efficiency.

## Introduction

Groundwater flow velocities and directions are important hydrogeological parameters for calculating the flux of groundwater and its solutes, and for determining the diffusion processes, areas of influence, and control measures related to groundwater pollution. The methods for obtaining groundwater flow velocities and directions can be divided into contact type and non-contact type methods. The former method is used to directly observe the real and dynamic state of the groundwater, while the latter can prevent the impact of the repeated applications of instruments or tracers on groundwater and reduce the damage to underground hydrogeological structures^1^. The conventional methods used to monitor groundwater flow velocities and directions include hydrogeophysical prospecting, the tracer method, and the injection method^1–8^, or a combination of multiple methods^3,9^. Of the contact monitoring methods, using a colloidal borescope based on microimaging allows the analysis and calculation of groundwater velocities and directions by tracking the motion of colloidal particles in groundwater. This method was established in the 1990s and represents an early stage set of groundwater sampling techniques based on colloidal borescope observations^10,11^. This method was then used to observe particle motion in monitoring wells^3^, before being gradually developed into a method for monitoring groundwater flow velocities and directions^12,13^. Finally, at the turn of this century, the method evolved into a technological system for monitoring groundwater flow velocities and directions^14^. The colloidal borescope enjoys the following advantages: (1) it applies advanced techniques to groundwater monitoring. The significant advancement in, and high sensitivity of, this method means that it can now be applied to the monitoring of extremely slow flow velocities, especially those of fracture water; (2) this method requires no tracers and does not produce pollutants; and (3) this method is easy to operate and is reusable, has a low cost and can be used to monitor both single wells and multi-well networks^15,16^. The colloidal borescope has been applied to regional hydrogeological surveys, the boundary delimitation of basin groundwater, the protection and management of important water sources, the disaster monitoring of mine groundwater, and major engineering construction projects^15,17–19^, mainly focusing on the investigation and monitoring of karst groundwater^20^. However, there are significant differences between the monitoring results produced using this method and the analytical and calculated results arising from use of the conventional hydrogeological methods, including: (1) significantly higher conventional infiltration rates converted from monitored groundwater flow velocities; (2) the swing of particles or probes as reflected by the symmetrical distribution (relative to the origin) of instantaneous velocities and directions; (3) significant differences between the flow velocities at the beginning and at the end of the monitoring cycle; and (4) significant differences between the flow velocities and directions obtained at the same position over different monitoring periods.

Reliability tests show that the laminar flow velocities observed using the colloidal borescope are consistent with the results obtained using the tracer method^3,21,22^. However, when the colloidal borescope is used to observe groundwater flow velocities and directions in heterogeneous aquifers, its monitoring results are close to the maximum velocity, meaning that a correction factor α = 1‒4 has been proposed for groundwater flow velocities in adjacent aquifers^3,12^. Similar to the particle imaging velocimetry of surface water^23^, the main factors affecting the monitoring of groundwater flow velocities and directions include the identifiability of the colloidal particles for tracing the water flow, the uniformity of the spatiotemporal distribution of the colloidal particles, the interference caused by the vertical groundwater flow to the horizontal flow in wells, and the turbulence of groundwater flow in heterogeneous aquifers, as well as the swing of monitoring probes in deep wells and the representativeness of any obtained observation data.

Bedrock fissure groundwater circulates in fissures under the constraint of solid boundaries. The motion elements of groundwater vary greatly in the fissure space due to the complex boundary geometries of the water-conducting tensile fissures in rocks. The irregular boundaries of the tensile fissures cause the size of the space to fluctuate greatly, easily forming eddy currents. As particles flow with groundwater, the superimposition of factors including eddy currents, collisions between particles and boundaries, and the Brownian motion of the particles themselves, make the motion paths of particles more complex and longer than those of groundwater. Moreover, since the flow velocity monitored in wells is the velocity when groundwater enters the large space of monitoring wells from the narrow fissure space, the instantaneous velocity monitored is relatively higher. Despite a certain randomness, it can be observed from the monitoring screen that colloidal particles in groundwater still move along a certain direction at a certain overall velocity^24^. The long-term monitoring results of colloidal particles in water generally represent the flow of groundwater in fissures^3,12^.

The establishment of directionally quantifiable horizontal flow is dependent on four parameters: borehole diameter, structure, permeability and the hydraulic gradient of the flowing feature^25^. The authors of this study hold that the cumulative and actual flow velocities obtained after restoring the particle motion trajectories can better represent the flow velocities and directions of fissure groundwater. In order to find a way that can reflect the flow velocity and direction of groundwater in underground aquifers under natural conditions, we established the concept of actual flow velocity based on instantaneous flow velocity, time-average flow velocity and cumulative flow velocity. The actual flow velocity refers to the flow velocity of groundwater in its natural state, which is an attempt to represent the flow velocity of groundwater in an aquifer over a long time scale. Therefore, we use the limit value of the flow velocity when the monitoring time tends to infinity as the actual flow velocity. To this end, the overall philosophy of this study was: (1) to restore the original state of groundwater flow in the absence of drilling disturbances through physical isolation using packers; and (2) to interpret the groundwater motion according to the particle motion using an improved algorithm based on big data technology. The specific scheme was as follows: first, monitoring probes were stabilized using deep-well devices for stabilization and catch, and the vertical flow interference was eliminated by employing layered hydraulic isolation, using packers. Second, the particle motion trajectories of groundwater were plotted, and the corresponding algorithms for the direction of motion and cumulative flow velocities were established, based on massive monitoring data of the flow velocities and directions of the fissure groundwater. Third, using the cumulative flow velocity vs. monitoring time curve-fitting equations, the limits of the cumulative flow velocities of groundwater as the monitoring time tends to infinity were calculated as the actual groundwater flow velocities at the various monitoring sites.

**Figure 1:**
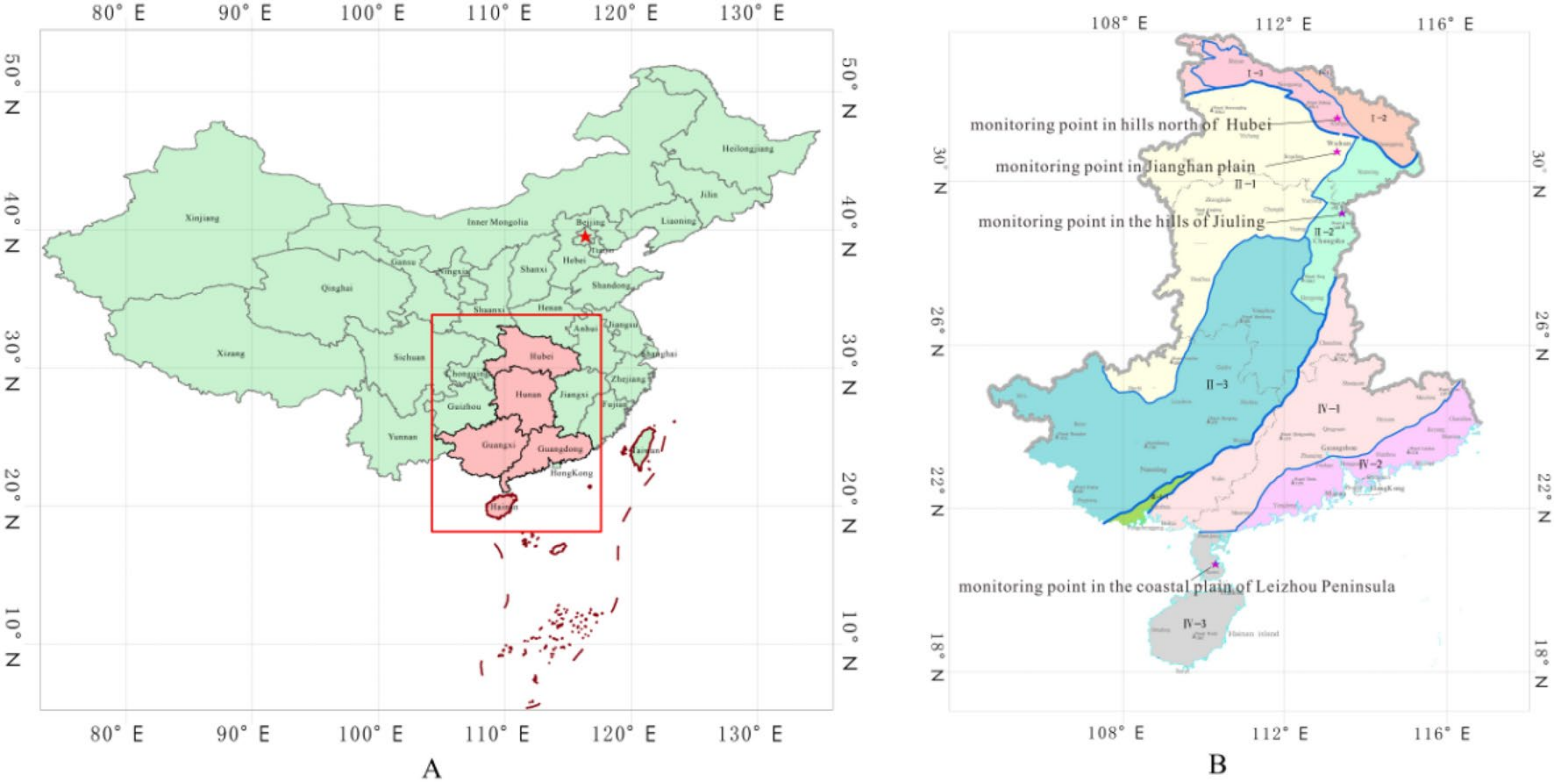
Location of the study area.

The study area is located in central-south China and covers five provinces, i.e., Hubei, Hunan, Guangdong, Guangxi and Hainan. It spans four major geographical units from north to south, namely the Dabie Mountains, the Mid-Yangtze River Plain, the Nanling-Jiuling Mountains, and the South China Coastal Hilly Plain, with a north–south length of > 1200 km and an altitudinal range of between 15 and 283 m. The study area stretches across three tectonic units, *i.e*., the Qinling-Qilian-Kunlun orogenic system (I), the Yangtze Block (II), and the Wuyi-Yunkai orogenic system (IV) (Fig. 1)^26,27^. A total of five monitoring wells were deployed by Table 1. The five wells represent different aquifers and different groundwater types. Well ZK01 is a limestone aquifer, representing dissolved fissure water. The lithology of well ZK106 is schist, which represents the tectonic fissure water in aquifer of metamorphic rocks. The DR01 well is granite, representing tectonic fissure water. Well HT35 is stomatal-almond basalt, representing pore fissure water. The lithology of well DR01 is granite, which represents tectonic fissure water. The lithology of HT35 well is porosity and almond basalt, which represents pore fissure water. The lithology of well PH33 is a sand aquifer, which represents loose rock pore water.

**Table 1:** Distribution of the wells monitoring groundwater flow velocities and directions.

Locations	Hills of northern Hubei	Jianghan Plain	Jiuling Mountains	Coastal plain on the Leizhou Peninsula	
Well number	Well ZK106	Well ZK01	Well DR01	Well HT35	Well PH33
Coordinates of the wells	114°1632′E 31°0917′N	114°4214′E 30°4581′N	113°9928′E 29°0830′N	110°4759′E 20°5631′N	110°3793′E 20°6384′N
Lithology of the monitored aquifer	Schist	Limestone	Granite	Basalt	Basalt
Depth of the well (m)	200	500	870	35	243
Altitude (asl) of the wellhead (m)	130	21	283	14.8	10.8
Altitude of the well base (m)	− 70	− 479	− 587	− 20	− 232.2
Groundwater depth (m)	8.6	1.4	2.52	7.1	18.1
Monitoring depth (m)	129	120, 134	120–236	13, 14, 15	21–30
Monitoring elevation (m)	1	− 113 ~ − 99	47 ~ 163	− 0.2 ~ 1.8	− 19.2 ~ − 10.2

## Data acquisition and processing

### Determination of monitoring positions

The locations of fissures to be monitored were determined using the GeoVISION™ VR borehole cameras manufactured by an United States-based company, Allegheny Instruments. The cable and underwater cameras were run into wells using a winch system to take videos inside boreholes, which were then transmitted to the surface in real time via the cable. Afterward, specific monitoring positions were selected by observing and inspecting the borehole integrity, borehole wall structure and well flushing, and by identifying the transparency of the water, the silts at the bottomhole, the distribution of the main rock interfaces, fracture zones, and silicified zones, the characteristic locations and occurrence of fracture planes, and the water flow condition. The preferred monitoring positions included gently inclined or horizontal fissures or fracture zones.

### Control of probe swing and reduction of superficial and vertical-flow interferences

Probe swing was prevented using a physical stabilization mechanism, and the superficial temperature, pressure interference and interlayer vertical-flow interference were blocked via hydraulic isolation, using packers. The monitoring probes were equipped with deep-well devices for stabilization and catch to prevent them from swinging. To simulate the original flow state of the groundwater, and to block the interference of superficial air temperatures and atmospheric pressure, as well as the vertical-flow interference caused by the pressure difference between aquifers and fissures at different depths in the wells, two measures were taken to obtain the actual water flow parameters of the target aquifers (fissures), viz*.*: (1) the monitoring windows were arranged under constant local temperature zones as far as possible; and (2) a high-resolution monitoring system based on layered hydraulic isolation was adopted. Specifically, the target aquifers (fissures) were arranged in the monitoring window sections. In this way, the vertical water flow was blocked using two (upper and lower) packers.

Therefore, we designed and manufactured the “Stabilization devices in deep wells” and “A layered isolation, high-fidelity monitoring system”. It tries to simulate the movement of groundwater in the fissure, to reduce the difference between the movement in the well and the movement in the crack pore. We have obtained the invention patent certificate from the State Intellectual Property Office of China.

### Monitoring of flow velocities and directions

This study employed a CS-1100-FG colloidal borescope with flux gate compass manufactured by Geotech Environmental Equipment, Inc., of the United States. More than 60 sets of this type of colloidal borescopes have been sold in China. The CS-1100-FG colloidal borescope is used to observe and trace the motion velocity and direction of colloidal particles in groundwater via a borescope, thus further determining groundwater flow velocity and direction. The data acquisition probes were installed in the window section of the high-resolution monitoring system, based on layered hydraulic isolation. AquaLITE Software It is only used to record the instantaneous velocity of groundwater, but not to calculate the average, cumulative, and actual velocity.

### Data preprocessing

#### Coordinate conversion

The groundwater velocity vectors monitored consisted of the flow speeds and the flow directions expressed as geographic azimuths, and were a set of parameters in polar coordinates. To avoid the error where (0° + 360°) averages 180°, and to ensure the convenience and accuracy of calculations, it was necessary to convert the polar coordinates into rectangular coordinates as follows:

1 $$\left\{\begin{array}{c}{V}_{x}=\rho \mathit{cos}\left\{\left(\theta -90\right)\frac{\pi }{180}\right\}\\ {V}_{y}=\rho \mathit{sin}\left\{\left(90-\theta \right)\frac{\pi }{180}\right\}\end{array}\right.$$

where *θ* is the azimuth of the flow direction, °; *ρ* is the flow speed, μm/s; *Vx* is the west–east component (W–E component) of the flow velocity, which is positive for an eastward direction and negative for a westward direction, μm/s; and *Vy* is the north–south component (N–S component) of the flow velocity, which is positive for a northward direction and negative for a southward direction, μm/s.

#### Reduction of data randomness and redundancy

Numerous monitorable particles appeared at the beginning of the monitoring process due to the interference caused by placing the probe, resulting in a particularly large data volume and severe data redundancy. As the standing time of the probes in wells increased, the data volume decreased significantly, leading to uneven data volumes. This study reduced data randomness and redundancy using averages in the same time interval. In other words, the statistical averages of the data in the same time interval were used as the flow velocities and directions within the time interval. Taking into account data continuity and consistency with time intervals for the monitoring of gravity solid tide and water pressure, this study adopted a time interval of 10 min, i.e., statistical averages were obtained at 10 min intervals, and taken as time average velocities.

#### Supplementation of data gaps

The colloidal borescope method is used to calculate groundwater flow velocities and directions by measuring the directions of the motion and velocities of the particles of appropriate sizes in the groundwater. If there is no monitorable particle in deep groundwater within a certain time interval, data gaps will occur. The time of any data interruption was controlled to within 5% of the monitoring time to ensure the continuity and consistency of the data. In this study, the time average velocity was calculated based on an interval of 10 min, and the continuous monitoring time was mostly over 10 h, during which 60 sets of data were obtained. The number of continuous interruptions was controlled at ≤ 3 for the time average velocity, that is, the continuous data interruption time was < 30 min. In cases where there were more than three continuous data interruptions (30 min), the data did not constitute continuous data, and the corresponding data gap was supplemented using the average of the four data values obtained before and after the data gap.

### Algorithm and plotting of particle motion trajectory

The motion trajectory of colloidal particles can be used to characterize the path of colloidal particles as they move within groundwater. The corresponding time average displacement and distance can be calculated using the time average velocities, after the reduction of data randomness and redundancy and coordinate conversion. The W–E component (*x*) and the N–S component (*y*) were accumulated separately. Making the endpoint of the previous time interval become the start point of the next time interval formed a continuous broken line, revealing the spatiotemporal patterns of colloidal particles at monitoring sites, and representing the general motion trajectory of colloidal particles. The rectangular coordinates of a point were calculated as follows:

2 $${\left\{\begin{array}{c}{x}_{n}=\sum_{i=1}^{n}{x}_{i}=\sum_{i=1}^{n}{{S\times v}_{i}}_{x}\\ {y}_{n}=\sum_{i=1}^{n}{y}_{i}=\sum_{i=1}^{n}{{S\times v}_{i}}_{y}\end{array}\right.}$$

where ${v}_{i}$ is the time average velocity, μm/s; ${{v}_{x}}_{i}$ is the W-E component of the time average velocity, which is positive for an eastward direction and negative for a westward direction, μm/s; ${{v}_{y}}_{i}$ is the N-S component of the time average velocity, which is positive for a northward direction and negative for a southward direction, μm/s; *S* is the duration of the calculated time average velocity, *s*; and *n* is a consecutive positive integer.

The particle motion trajectory was plotted by connecting points $\left({x}_{1}{, y}_{1}\right), \left({x}_{2}{, y}_{2}\right), \left({x}_{3}{, y}_{3}\right)\dots \left({x}_{i}{, y}_{i}\right) \dots \left({x}_{n}{, y}_{n}\right)$ using the data analysis software V1.0 program for deep groundwater migration.

### Analysis and calculation of flow directions

The overall flow direction of groundwater was analyzed based on the particle motion trajectory; the specific direction was usually calculated using the trendline equation. When data changed greatly and the trendline derived from the trajectory could not represent the flow direction, the endpoint coordinates of particles were used to calculate the flow direction.

#### Trendline equation method

The preprocessed data were plotted, and the trendline equation was then generated from the plot. Finally, the included angle between the trendline and the *x*-axis was calculated based on the slope of a straight line in the trendline equation before being converted into the azimuth.

#### Endpoint coordinate algorithm

Connecting the start point and endpoint of the monitoring formed a straight line. Next, the included angle between the straight line and the *x*-axis was calculated using the coordinates of both the start point and the endpoint. Finally, the included angle was converted into the azimuth:

3 $$\alpha =A-\frac{180}{\pi }\mathit{arctan}\left(k\right) \left\{\begin{array}{l}x>0,A=90\\ x=0,y<0,\alpha =180\\ x=0,y>0,\alpha =360\\ x<0,A=270\end{array}\right.$$

where *k* is the slope, and *A* is a constant.

### Calculation of the cumulative flow velocity and cumulative flow direction of groundwater

According to the motion trajectory of a particle, the particle position is $\left({x}_{t}{, y}_{t}\right)$ at time* t*, meaning that the distance *L* between the particle and the origin is:

4 $$L=\sqrt{{{x}_{t}}^{2}+{{y}_{t}}^{2}}$$

The cumulative flow velocity $({v}_{c})$ of groundwater is defined as the ratio of the total displacement *L* of the particle to the time interval (*t)*. The equation for cumulative flow velocity is:

5 $${v}_{c}=\frac{L}{t}=\frac{\sqrt{{{x}_{t}}^{2}+{{y}_{t}}^{2}}}{t}$$

The method for calculating the cumulative flow direction is shown in Eq. (3), and the equation for calculating slope ($$k)$$ is:

6 $$k=\frac{{y}_{t}}{{x}_{t}}$$

The cumulative flow velocity represents the overall velocity of a particle traveling away from the origin during the calculation period. A lower velocity corresponds to a shorter distance from the origin for a certain time interval, and vice versa. The cumulative flow velocity and direction of particles in water can effectively represent the state of flow of the groundwater at monitoring sites.

### Calculation of the actual flow velocity of groundwater

The cumulative flow velocity of groundwater over a longer monitoring time was closer to its actual flow velocity at the monitoring sites. Therefore, the actual flow velocity of groundwater could be obtained using infinite-time monitoring. The cumulative flow velocity *vs.* monitoring time curves were plotted using the cumulative flow velocities recorded at different times during the monitoring process. The curve-fitting equations were then established, each of which used time* t* as the independent variable, and the cumulative flow velocity *v*_*c*_ as the dependent variable. The limit of the equation as time tends to infinity could then render the actual flow velocity, thus:

7 $${v}_{a}=\underset{t\to \infty }{\mathit{lim}}{v}_{c}(t)$$

where ${v}_{a}$ is the actual flow velocity, μm/s; and ${v}_{c}$ is the cumulative flow velocity, μm/s.

**Figure 2:**
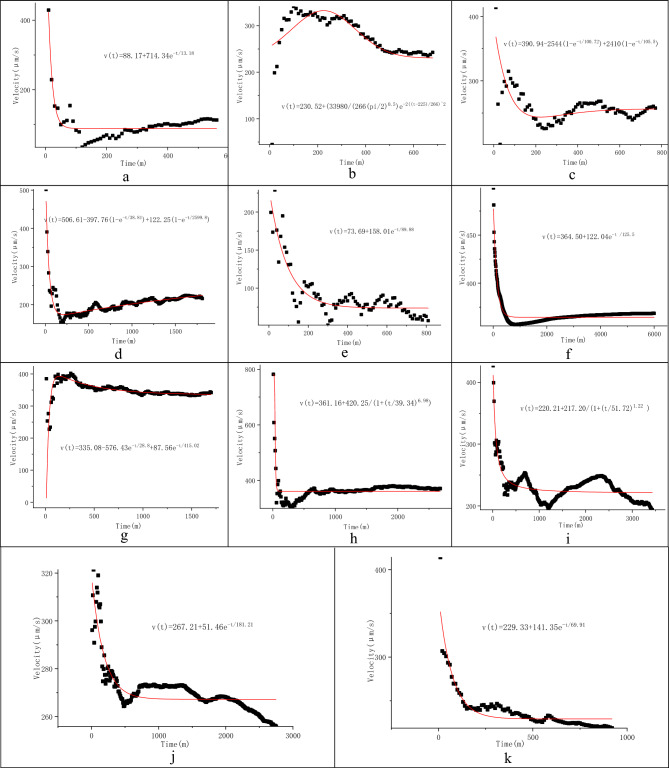
Cumulative flow velocity *vs.* monitoring time curves in Well DR01 (**a**–**e**), Well ZK01 (**f**–**i**), and Well ZK106 (**j**, **k**) (the monitoring depths and dates are in parentheses).

**Figure 3:**
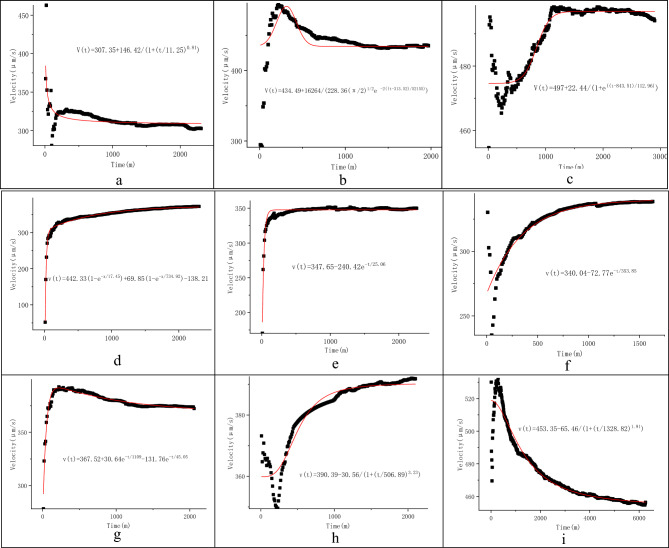
Cumulative flow velocity vs. monitoring time curves in Well PH33(**a**–**c**) and Well HT35(**d** ~ **i**) (the monitoring depths are in parentheses).

**Figure 4:**
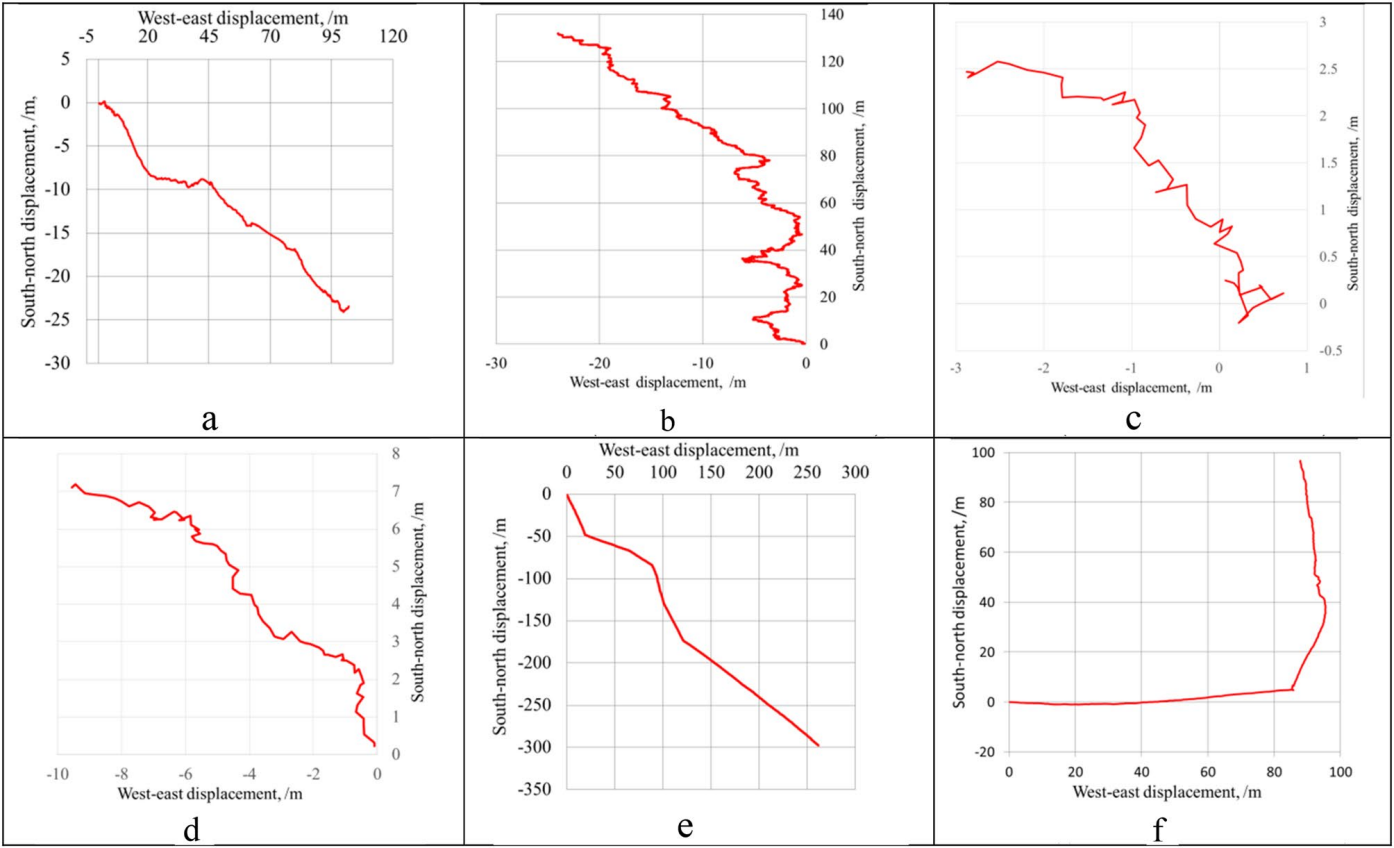
The motion trajectory of particles in the groundwater (the monitoring dates and depths are in parentheses).

In line with the monitoring and calculation results of the flow velocities and directions of fissure groundwater in the study area, the cumulative flow velocity *vs.* monitoring time curves were divided into four types: (1) L shaped curves, representing rapid changes in the flow velocity from high to low, before fluctuating slowly, and tending towards a stable value as time increases (Fig. 2a,c,d,e,f,h,i); (2) Γ shaped curves, representing rapid changes in the flow velocity from low to high, before fluctuating slowly, and tending towards a stable value (Fig. 3d,e,g); (3) V^—^ shaped curves, representing rapid changes in the flow velocity from high to low with time, then before increasing significantly, and tending towards a stable value (Fig. 3c,f,h) and (4) Λ_ shaped curves, denoting a rapidly changing flow velocity from low to high, before decreasing significantly, and finally tending towards a stable value (Fig. 3i). Analyses of the shapes of these curves indicated that it was possible to obtain the limits of the flow velocities.

The actual flow velocity was calculated as follows: (1) the cumulative flow velocities of different monitoring time intervals were calculated using the data obtained from the continuous monitoring process, without artificial disturbance and data interruption; (2) the cumulative flow velocity *vs.* monitoring time curves were plotted; (3) the curve-fitting equations, with time *t* as the independent variable and cumulative flow velocity *v*_*c*_ as the dependent variable, were established; and (4) the curve-fitting equations were analyzed, and then the limit of the cumulative flow velocity as the independent variable (monitoring time) tends to infinity was calculated as the actual flow velocity.

To ensure that the limit could be obtained, the exponential or logarithmic equation model was usually selected to render the curve-fitting equation. The curve-fitting equations with residual errors decreasing with time were preferred when residual errors varied greatly. Moreover, *R*^2^ had to approach 1 and the variance had to be low, as far as possible. The Levenberg Marquardt was preferred as the iterative optimization algorithm. The orthogonal distance regression (ODR) based iterative optimization was selected when *R*^2^ was < 0.8, the residual error was too large, or the limit could not be obtained.

The “Deep Groundwater Migration Data Analysis Software” was used to data preprocessing, calculation and drawing of glue particle movement trajectory, analysis and calculation of flow direction, and calculation of groundwater cumulative flow rate. In 2020, we programmed and developed this computing software, which was upgraded to V2.0 in 2023. We have obtained the copyright certificate certified by the State Intellectual Property Office of China.

Both the determination and mathematical statistics of the curve-fitting equations in this study were completed using the Orange 2018 software.

## Results

In this study, numerous instantaneous velocities were obtained from monitoring, and the time average velocity was calculated every 10 min. Then, the cumulative flow velocities every 10 min during the whole monitoring process were calculated from the accumulation of the time average velocities. The three types of flow velocities were statistically analyzed. The cumulative flow velocity *vs.* monitoring time curves were then plotted and the curve-fitting equations were then established using the cumulative flow velocity data series. Finally, the limit as the monitoring time tended to infinity was calculated as the actual flow velocity. Only one actual flow velocity value was to be obtained for each continuous monitoring process. The following is the calculation of groundwater velocity and flow in five monitored wells in the four regions.

### Well ZK106, in the hilly area of northern Hubei Province

Well ZK106 is located in a low-mountainous, hilly area, where mountains have strikes of NW and NNW and peaks of 300‒600 m asl. The water-bearing formations in the area are composed of the metamorphic rocks of the Proterozoic Hong'an Group, which are mainly composed of sericite-quartz schists. These rocks have well-developed schistosity and gneissosity and locally-developed fractures, which, however, are frequently filled with weathered materials. The weathered layers generally have a thickness of 20 ‒ 30 m. This area has poor water yield properties, very small water flow quantity, and few springs, with a flow quantity generally of < 3 m^3^/day^28^ and a single-well exploitation volume of domestic wells of 2‒5 m^3^/day. The groundwater in this area is mainly recharged by atmospheric precipitation, but the infiltration recharge is very low. Well ZK106 revealed artificial fills at depths of 0‒39 m, which were cased using PVC plate tubes, and also revealed schists at a depth of 39‒200 m, with steel filter tubes installed at depths of 39‒120 m. This well was monitored from 13:26 on September 22, 2019 to 09:37 on September 26, 2019, obtaining 124,259 pieces of data in total.

As shown by the analytical results of the curve-fitting equations of the particle motion trajectories, the groundwater at Well ZK106 had a general flow direction of 102.18°, i.e., a WNW-ESE trend (Fig. 4a). The results of the time average velocity were as follows: (1) its maximum was 7.8 times its minimum, indicating a huge difference; (2) its N‒S and W‒E components had similar maxima, but greatly different minima, with the minimum of the W‒E component almost 3 times that of the N‒S component; and (3) the W‒E component was positively correlated with the resultant velocity, with a correlation coefficient of 0.92, but the N‒S component was negatively correlated with the resultant velocity, with a correlation coefficient of -0.38. The maximum instantaneous velocity was 21 times its minimum. For the actual flow velocity, its maximum was 1.2 times its minimum. In terms of the cumulative flow velocity, its maximum was 1.3 times its minimum, and the correlations between its resultant velocity and the W‒E and N‒E components were similar to those of the time average velocity (Table 2).

**Table 2:** The velocities and directions of fissure water in the study area (Unit: μm/s).

Well	Type	Sum	Min	Max	Median	M. V	SD	C. V
Well ZK106, Hills in northern Hubei	*Vi*	124,259	71.3	1499.3	354.8	423.63	242.23	0.57
	*Vt*	552	123.75	968.13	292.74	339.25	126.29	0.37
	*Vc*	552	255.44	336.93	274.8	292.19	28.41	0.1
	*Va*	2	229.33	267.21	248.27	248.27	–	–
Well ZK01, Jianghan plain	*Vi*	48,310	84.1	1498.2	453.1	504.31	217.47	0.43
	*Vt*	1542	55.34	1180.62	426.5	432.16	133.63	0.31
	*Vc*	1542	193.26	498.94	281.29	304.78	69.99	0.23
	*Va*	4	220.21	364.5	348.12	320.24	58.86	0.18
Well DR01, Jiuling Mountains	*Vi*	175,663	11.6	1596.8	584.7	635.17	224.95	0.35
	*Vt*	522	14.79	1023.5	334.55	340.26	162.56	0.48
	*Vc*	522	34.46	204	501.76	188.3	77.4	0.41
	*Va*	5	73.69	285.1	230.52	186.82	88.29	0.47
Well HT35, Leizhou Peninsula	*Vi*	213,567	69.8	1200	416.7	415.42	163.53	0.39
	*Vt*	1842	46.57	958.8	397.58	418.85	94.23	0.23
	*Vc*	1698	51.83	388.86	346.45	348.72	20.69	0.06
	*Va*	6	340.04	390.39	370.75	378.82	37.23	0.09
Well PH33, Leizhou Peninsula	*Vi*	30,835	49.1	1994.4	430.1	453.9	221.22	0.49
	*Vt*	722	30.24	1135.3	435.19	429.06	103.73	0.24
	*Vc*	718	303.37	498.67	423.24	412.92	76.25	0.18
	*Va*	3	307.35	497	434.49	412.95	78.91	0.19

*V*_*i*_ instantaneous flow velocity, *V*_*t*_ time average velocity, *V*_*c*_ cumulative flow velocity, Va actual flow velocity, *Min.* minimum, *Max.* maximum, *M.V.* Mean value, *S.D.* standard deviation, *C.V.* coefficient variation.

Both the instantaneous velocity and the time average velocity had unimodal and skewed statistical histograms, with a maximum range of 200‒300 μm/s. The cumulative flow velocity was represented by a bimodal statistical histogram, with two maxima of 260‒270 μm/s and 320‒330 μm/s. The W‒E component of the cumulative flow velocity also rendered a bimodal statistical histogram, but its N‒S component was unimodal. The distribution of the cumulative flow velocity was therefore more closely related to its W‒E component.

This well was monitored twice at a depth of 129 m, yielding two cumulative flow velocity *vs.* monitoring time curves in an L-shape (Fig. 2j,k). The equations fitting these two curves were established using the ExpDec1 mathematical model and the ODR-based iterative optimization. They had an *R*^2^ value of 1.00, indicating a high degree of fit. The actual flow velocity of groundwater at the monitoring site was calculated to be 267.21 μm/s and 229.33 μm/s, with an average value of 248.27 μm/s (Table 3).

**Table 3:** The actual flow velocity of fissure water fitted using the equation limit method.

Type of groundwater	Depth (m)	Duration (m)	Model	Curve-fitting equation	*Va *(μm/s)	*R*^2^
Water in sand layer	13	2310	Logistic	*V(t)* = *307.35* + *146.42/(1* + *(t/11.25)*^*0.81*^*)*	307.35	0.99
	14	1970	Gauss	*V(t)* = *434.49* + *16,264/(228.36 *$\sqrt{\frac{\pi }{2}}$* e*^*−2((t−313.52)/52155*^*)*	434.49	0.99
	15	2900	Boltzmann	*V(t)* = *497* + *22.44/(1* + *e*^*((t−843.51)/112.96)*^*)*	497.00	1
Fissure water in vesicular basalts	21	2320	ExpAssoc	*v(t)* = *442.33(1 − e*^*−x/17.45*^*)* + *69.85(1 − e*^*−x/734.92*^*) − 138.21*	373.97	0.99
	23	2260	ExpDec1	*v(t)* = *347.65–240.42e*^*−t/25.06*^	347.65	0.93
	25	1630	ExpDec1	*v(t)* = *340.04–72.77e*^*−t/353.85*^	340.04	0.83
	27	1980	ExpDec2	*v(t)* = *367.52* + *30.64e*^*−t/1109*^*–131.76e*^*−t/45.05*^	367.52	0.96
	29	2100	Logistic	*v(t)* = *390.39–30.56/(1* + *(t/506.89)*^*3.23*^*)*	390.39	0.92
	30	6260	Logistic	*v(t)* = *453.35–65.46/(1* + *(t/1328.82)*^*1.91*^*)*	453.35	0.94
Fissure water in granites	120	560	ExpDec1	*v(t)* = *88.17* + *714.34e*^*−t/13.18*^	88.17	0.83
	131	680	Gauss	*v(t)* = *230.52* + *(33,980/(266(pi/2)*^*0.5*^*)e*^*−2((t−225)/266)^2*^	230.52	0.98
	141	770	ExpAssoc	*v(t)* = *390.94–2544(1 − e*^*−t/100.72*^*)* + *2410 (1 − e*^*−t/105.5*^*)*	256.66	0.99
	228	1810	ExpAssoc	*v(t)* = *506.61–397.76(1 − e*^*−t/38.81*^*)* + *122.25 (1 − e*^*−t/2599.8*^*)*	285.10	0.89
	236	810	ExpDec1	*v(t)* = *73.69* + *158.01e*^*−t/89.88*^	73.69	0.80
Fissure water in limestone	134	6000	ExpDec1	*v(t)* = *364.50* + *122.04e*^*−t/125.5*^	364.50	1
	134	1700	ExpDec2	*v(t)* = *335.08–576.43e*^*−t/28.8*^ + *87.56e*^*−t/415.02*^	335.08	0.99
	134	26,800	Logistic	*v(t)* = *361.16* + *420.25/(1* + *(t/39.34)*^*6.98*^*)*	361.16	1
	120	3430	Logistic	*v(t)* = *220.21* + *217.20/(1* + *(t/51.72)*^*1.22*^*)*	220.21	1
Fissure water in schists	129	2750	ExpDec1	*v(t)* = *267.21* + *51.46e*^*−t/181.21*^	267.18	1
	129	920	ExpDec1	*v(t)* = *229.33* + *141.35e*^*−t/69.91*^	229.33	1

### Well ZK01, on the Jianghan plain

Well ZK01 is located in an area of plains, rivers and lakes, where the terrain undulates gently, with a gradient of slope of < 3°. This area has an altitude of ~ 20‒30 m asl. Most of this area is covered by Quaternary strata, with underlying Paleozoic-Cenozoic strata. The groundwater in this area consists of the pore water in unconsolidated rocks, the fissure-pore water in clastics, fissure water in the solution-enlarged fractures in limestones, and fissure water in weathered bedrock. Fissure water in the solution-enlarged fractures in limestones can be found in the solution-enlarged fractures of the Triassic Jialingjiang Formation aquifer, the Carboniferous-Permian Qixia Formation aquifer, and the Qijiaoshan Formation aquifer, the last one being a formation that belongs to the Proterozoic Hong'an Group. These water-bearing formations are mainly composed of limestones, dolomites and marbles. They have extremely uneven water yield properties as a result of their lithologies, fault structures, and degree of karst development, with a single-well water yield of 141‒878.00 m^3^/day and moderate-abundant water quantities. The groundwater in the hilly area’s piedmont is largely dominated by modern water, which flows from the hinterland of the plain to the discharge areas of the Hanjiang and Yangtze rivers. The groundwater ages ranged from a few hundred years to 6000 year; the water circulates and is slowly displaced^29^. The groundwater is mostly characterized by simple hydrochemical facies of HCO_3_‒Ca‒Na, HCO_3_‒Ca‒Mg and HCO_3_‒Ca, a pH of 6.1‒8.4, a total dissolved solids (TDS) content of 30‒830 mg/L, and a total hardness of 21‒675 mg/L^30,31^. Well ZK01 revealed artificial fills at depths of 0‒3.9 m, yellowish-brown and gray clays at depths of 3.9‒8.6 m, and limestones at depths of 8.9‒500 m. This well was cased using stainless steel tubes at depths of 0‒12 m, and the borehole walls were exposed at depths of 12‒500 m. This well was monitored from June 19 to June 25, 2019, and from December 19 to December 24, 2019, obtaining 48,310 pieces of flow velocity and direction data in total.

As shown by the particle motion trajectory of groundwater at Well ZK01, the groundwater had a flow direction of 352.12°, i.e., a SSE-NNW trend overall, with the N‒S velocity component much greater than the W‒E velocity component (Fig. 4b). The instantaneous velocity maxima (minima) of its W‒E and N‒S velocity components had equivalent absolute values but opposite directions; its resultant velocity had a maximum 17.8 times its minimum. The time average velocity of groundwater at this well was as follows: (1) its resultant velocity had a maximum 21 times its minimum; (2) its W‒E component had an approximate maximum and minimum, but the negative W‒E component had higher absolute values than the positive W‒E component, indicating that the particles, along with the groundwater, exhibited a small W‒E displacement and had a westward displacement overall; and (3) its N‒S component had a maximum 2.3 times the minimum, and the positive N‒S component had far higher absolute values than the negative N‒S component, indicating a significant northward displacement of the groundwater. The cumulative flow velocity of the groundwater at this well was as follows: (1) the maximum resultant velocity was 2.2 times the minimum; (2) it had a negative W-E component, for which the absolute maximum was significantly greater than the absolute minimum, indicating a westward flow of groundwater; and (3) it had a positive N‒S component, for which the maximum was 2.6 times the minimum, indicating a northward flow of groundwater (Table 2). The actual flow velocity had an average value of 320.24 μm/s; its maximum was 1.66 times its minimum, indicating a large variation.

Both the instantaneous velocity and the time average velocity were represented by unimodal statistical histograms. The statistical histogram of the former was slightly skewed, with a maximum range of 400‒600 μm/s, and that of the latter was symmetrical, with a maximum range of 400‒500 μm/s. The cumulative flow velocity rendered a bimodal statistical histogram, with maximum value ranges of 220‒230 μm/s and 360‒380 μm/s. Its N‒S component also rendered a bimodal statistical histogram, while its W‒E component appeared unimodal, indicating that the cumulative flow velocity is mainly controlled by its N‒S component.

This well was monitored three times at a depth of 134 m in June and December, 2019, yielding three cumulative flow velocity *vs*. monitoring time curves. Moreover, it was also monitored once at a depth of 120 m, yielding one cumulative flow velocity *vs.* monitoring time curve. The four curves consisted of a Γ-shaped curve (Fig. 2i) and three L-shaped curves (Fig. 2g,f,h). Of these, the L-shaped curve corresponding to a depth of 120 m showed that velocity varied in a sinusoidal pattern in its late stages (Fig. 2i). The equations fitting these curves were established using the ExpDec and Logistics mathematical models and the ODR-based iterative optimization algorithm. Their R^2^ values were 0.99‒1.00, indicating a reasonable degree of fit. The actual flow velocity of the groundwater was calculated to be 220.21‒364.50 μm/s, with an average value of 320.24 μm/s, for which the maximum was 1.7 times the minimum (Table 3).

### Well DR01, in the Jiuling mountains

Well DR01 is located in the tectonically denuded low-mountainous, hilly area to be found in the northwestern Jiuling Mountains. This area lies at an altitude of 200 ‒600 m asl (maximum: 1,511 m asl), a relative height difference of 70 ‒ 300 m, and valley plain widths of 200‒300 m. The bedrock in this area consists of biotite monzogranites, which were formed by crustal remelting and reconstruction during the Late Jurassic^32^; these bedrocks are typical of a syn-collisional and volcanic arc tectonic setting^33^. The groundwater in this area primarily occurs in developed, weathered fissures and structural fractures. The springs in this area have a flow rate of 0.1–0.45 L/s in the dry season, and a high flow rate in zones with structural fractures, where hot springs have a flow rate of up to 1.877 L/s. The groundwater in this area contains principally HCO_3_^−^ + SO_4_^2−^‒K^+^ + Na^+^ hydrochemical facies. Well DR01 revealed loose deposits at depths of 0‒8.9 m and biotite monzogranites at depths of 8.9 m to the bottom of the well. This well was cased using steel tubes with a diameter of 219 mm at depths of 0‒60 m, and the borehole wall was exposed below 60 m. This well was monitored from July 19 to July 27, 2019, and 530,947 pieces of data in total were obtained.

As shown by the particle motion trajectory of the groundwater, the groundwater at depths of 120 m, 141 m and 236 m in Well DR01 had flow directions of 306.99°, 306.02° and 316.94°, respectively, i.e., a SE‒NW direction (Fig. 4c,d). The instantaneous velocity maxima and minima of its W‒E and N‒S components had equivalent absolutes values but opposite directions. As for its resultant velocity, the maximum was 137 times the minimum. The time average velocity of the groundwater at this well was as follows: (1) the combined flow velocity maximum was 69 times the minimum; (2) the W‒E component minimum was − 1.5 times the maximum, indicating that the particles have a generally westward displacement; and (3) the N-S component maximum was − 1.4 times the minimum. The cumulative flow velocity of the groundwater at this well was as follows: (1) the resultant velocity maximum was 5.9 times the minimum; (2) the W‒E component maximum was − 1.2 times the minimum; and (3) the N‒S component maximum was − 7.3 times the minimum. The actual flow velocity had an average value of 186.82 μm/s, and its maximum was 3.9 times its minimum (Table 2).

The instantaneous velocity was represented by an unimodal and skewed statistical histogram, with a maximum value of 400–500 μm/s. The time average velocity rendered an unimodal and nearly normal statistical histogram, with a maximum value of 200–300 μm/s. The cumulative flow velocity was represented by a bimodal statistical histogram, with maxima of 50–100 μm/s and 200–250 μm/s. Moreover, its W‒E component was unimodal, while its N‒S component was bimodal, indicating that the groundwater flow velocity at Well DR01 is principally controlled by the N-S component.

Five cumulative flow velocity *vs*. monitoring time curves corresponding to five monitoring depths were plotted. They consisted of L-shaped curves for depths of 120 m, 141 m, 228 m and 236 m (Fig. 2a,c,d,e), a Γ-shaped curve corresponding to a depth of 131 m (Fig. 2b). The equations fitting these five curves were then established using the ExpDec1, Gauss, and ExpAssoc models, employing the Leverage Marquardt optimization algorithm (for the curves corresponding to depths of 120 m, 131 m and 141 m) and the ODR-based iterative optimization algorithm (for the curves corresponding to depths of 228 m and 236 m). The curve-fitting equations had *R*^2^ values of 0.80‒0.99, indicating a high degree of fit. The actual flow velocity was calculated to be 73.69‒285.10 μm/s, with an average value of 186.82 μm/s, for which the maximum was 3.9 times the minimum (Table 3).

### Wells PH33 and HT35, on the Leizhou Peninsula

Monitoring wells PH33 and HT35 are located in the coastal plain of the Leizhou Peninsula. This area has widely distributed Quaternary volcanic rocks and sand layers, which overlie a Zhanjiang Formation platform. There are two aquifers in this area, they are composed of sand layers and basalts formed by multiple volcanic eruptions, the majority of which occurred at 180 ka^34^. During the intervals between eruptions, weathered, residual, cohesive soils of a considerable thickness were formed on the top of basalts. As a result, vesicular basalts and residual soils appear alternately, forming 2‒3 layers of vug-fissure aquifers with a thickness of 3‒150 m. The aquifers have poor-rich water yield properties, with single-well water yield of 12.1‒4958 m^3^/day, and a spring flow quantity of 0.1‒24.886 L/s. The groundwater in the area is recharged by atmospheric precipitation through seepage or volcanic plumbing^35^. The runoff in the area flows radially, with volcanic cones as centers, creating favorable runoff conditions. The groundwater mainly contains a principally HCO_3_-Mg·Ca (Ca·Mg) hydrochemical facies , and a TDS content of 120‒310 mg/L^36,37^. The aquifers in the area consist of the basalts of the Middle Pleistocene Shimao Formation (Q_s_), with vesicular structures and locally-developed fissures. The overlying strata are composed of the yellow‒grayish yellow‒grayish white fine-grained (FG) sandy layers of the Holocene Xinliao Formation (Q_xi_), and the underlying strata consist of variegated clays interbedded with silty clays of the Middle Pleistocene Zhanjiang Formation (Q_z_).

#### Well HT35

The aquifer is composed by stomatal and almond basalt. The filter tube was placed in the basalt aquifer in the well. Seamless steel tubes were used for isolating the water in the upper and lower non-basalt rocks.

The trendline equation of the groundwater at Well HT35 was established using the motion trajectory of the particles in the groundwater (Fig. 4e). Next, the groundwater flow direction was calculated to be 111.97°‒165.85°, with an average value of 141.46° (Table 4). The instantaneous velocity of the groundwater was as follows: (1) its maximum was 17 times its minimum; (2) the W‒E component of the instantaneous velocity had maximum and minimum absolute values that were similar but opposite in direction, and its eastward velocity was slightly higher than its westward velocity; and (3) the N‒S component had similar maximum and minimum absolute values, but opposite directions, and its northward velocity was slightly lower than its southward velocity. In terms of the time average velocity, its maximum was 20 times its minimum, its eastward component was 3.8 times its westward component, and its southward component was 6.3 times its northward component. Its cumulative flow velocity maximum was 7.5 times its minimum, its eastward component was 12 times its westward component, and its southward component was 7.3 times its northward component. The groundwater at Well HT35 had an actual flow velocity of 340.04‒390.39 μm/s, with an average value of 378.82 μm/s (Table 2).

**Table 4:** Calculation of groundwater flow direction in wells PH33 and HT35.

Depth (m)		Curve-fitting equation	*R*^*2*^	*K*	Direction (°)
Well PH33	15	y = 0.0691x − 2E+06	0.8209	0.0691	86.05
	13	y = 3.2913x + 1715.1	0.8624	3.2913	16.90
	14	y = − 9.2893x + 5500.5	0.9556	− 9.2893	353.86
Well HT35	21	y = − 2.4526x + 571.91	0.9981	− 2.4526	157.82
	23	y = − 0.4035x-283.48	0.9994	− 0.4035	111.97
	25	y = − 0.7237x + 831.58	0.9995	− 0.7237	125.89
	27	y = − 3.966x + 5261.8	0.9906	− 3.966	165.85
	29	y = − 2.1741x-352.96	0.9997	− 2.1741	155.30
	30	y = − 0.8982x + 4973.4	0.9995	− 0.8982	131.93

The instantaneous velocity was represented by a complex, multimodal, and skewed statistical histogram, with maxima of 300‒350 μm/s and 400‒450 μm/s. The time average velocity rendered an unimodal and normal statistical histogram, with a maximum of 350‒400 μm/s. The cumulative flow velocity was represented by a bimodal statistical histogram, with maxima of 295‒315 μm/s and 335‒355 μm/s. Its W‒E and N‒S components both rendered a bimodal statistical histogram.

There are six cumulative flow velocity *vs.* monitoring time curves of three types were plotted, namely the Γ-shaped curves corresponding to depths of 21 m, 23 m and 27 m (Fig. 3d,e,g), the V-shaped curves corresponding to depths of 25 m and 29 m (Fig. 3f,h), and the Λ-shaped curves corresponding to a depth of 30 m (Fig. 3i). Curve-fitting equations were then established using the ExpAssoc, ExpDec and Logistic models. Their *R*^2^ values were 0.83‒0.99, indicating a high degree of fit. The actual flow velocity of the groundwater was calculated to be 340.04 ‒ 390.39 μm/s, with an average value of 378.82 μm/s (Table 3).

#### Well PH33

The aquifer is composed of a Quaternary sand layers. The filter tube was placed in the sand aquifer in the well. Use a seamless steel pipe to isolate the non-aquifer layer.

The groundwater at monitoring well PH33 has a NE flow direction overall, although its direction varies greatly (Fig. 4f, Table 4). The maximum instantaneous velocity of the groundwater was 40 times its minimum, its eastward component was 1.1 times its westward component, and its northward component approached its southward component in value. The maximum time average velocity of the groundwater at Well PH33 was 37 times its minimum, its westward component was 1.3 times its eastward component, and its northward component was 2.6 times its southward component. In terms of the cumulative flow velocity of the groundwater at this well, its maximum was 1.6 times its minimum, its northward component was 2.4 times its southward component, and its W-E component was positive, indicating an eastward groundwater flow. The actual flow velocity of the groundwater ranged between 307.35 and 497.00 μm/s, with an average value of 412.95 μm/s (Table 2).

The instantaneous velocity appeared as an unimodal and skewed statistical histogram, with a maximum value of 400‒500 μm/s. The time average velocity rendered an unimodal and nearly normal statistical histogram, with a maximum value of 400‒500 μm/s. The cumulative flow velocity appeared as a bimodal statistical histogram, with two maxima of 300‒320 μm/s and 480‒500 μm/s at both ends. Its N‒S and W‒E components both appeared as skewed statistical histograms, thus jointly controlling the bimodal characteristics of the cumulative flow velocity.

After continuity screening, three cumulative flow velocity *vs.* monitoring time curves corresponding to three monitoring depths were plotted, namely an L-shaped curve (Fig. 3a), a Γ-shaped curve (Fig. 3b), and a V-shaped curve (Fig. 3c), which corresponded to depths of 13 m, 14 m and 15 m, respectively. Afterward, curve-fitting equations were established based on the Boltzmann, Gauss and Logistic models, respectively, using the ODR-based iterative optimization algorithm (Fig. 3a‒c, Table 3). These curve- fitting equations had *R*^*2*^ values of 0.99‒1, indicating a degree of fit. The actual flow velocity of the groundwater was calculated to be 307.35‒497.00 μm/s, with an average value of 412.95 μm/s.

## Discussion

### The permeability coefficient of the aquifer calculated by monitoring is compared with that of pumping test

According to Darcy's law, the factors affecting the flow velocity of groundwater in the aquifer are the Head pressure and permeability coefficient. The permeability coefficient is determined by the fissure or porosity and structure of the aquifer. It is a parameter reflecting the basic physical properties of the aquifer. The pumping experiment is to produce the large Head pressure under the human intervention, and the capsule hole mirror is the observation flow rate under the natural Head pressure, and the Head pressure of the latter is often significantly smaller than the former. Therefore, in the same aquifer, the groundwater flow velocity observed by the two methods are quite different. However, Comparing the coefficient of aquifer permeability obtained from the two different methods can test the deviation of both results.

#### Calculation of permeability coefficient

##### The permeability coefficient calculated by the pumping test

1. Calculation of the permeability coefficient of confined water aquifer.

The aquifer in Well DR01 is a fractured zone in granite, there are 4 fracture zones in the drilling core, all of which are obviously broken, and the total thickness is 7.5 m, so M = 7.5 m.

The aquifer of well PH33 is the sand layer of the Quaternary Zhanjiang Formation, there are two layers, the thickness is 8.73 m, covered with aquiclude, which is clay and the thickness is 10 m. Between the two aquifers is a clay aquiclude.

No pumping test was carried out in well ZK106, so we chose the permeability coefficient and fracture rate calculated in well ZK19106 nearby. This well was 105 m deep and the pumping test was completed. The aquifer was proterozoic schist, the same as well ZK106, covered with silty clay and gravel clay waterproof layer with a thickness of 38 m.

ZK01 well did not do pumping test. We chose the permeability coefficient and fracture rate calculated by the nearby well S5, which was 60.6 m deep, and completed the pumping test. The aquifer is the same as well ZK01 and is a Triassic limestone with a thickness of 51.1 m. It is covered with silty clay and is a water-proof layer with a thickness of 9.5 m.

The four Wells are all complete Wells with confined water as the groundwater type, and steady flow pumping tests have been carried out. The permeability coefficient was calculated by Jules Dupuit equation, and the influence radius was calculated by Gittelt equation.

8 $$k=\frac{0.336Q}{MS}lg\frac{R}{r}, R=10S\sqrt{K}$$

where, *k* is the permeability coefficient, m/d; *Q* is the amount of groundwater pumped out, m^3^/d; *M* is the thickness of the aquifer, m; *s* is the depth by which the water level drops, m; *R* is the influence radius of pumping, m; *r* is the radius of the pumping hole, m.

② Calculation of the permeability coefficient of submersible aquifer

The aquifer of well HT35 is quaternary stomatal almond basalt of 12.76 m thickness; covered with quaternary sand layer of 5 m thickness. Groundwater is diving, the well is a complete well, and the stable flow pumping test was carried out. The permeability coefficient is calculated by the Jules Dupuit equation and the influence radius is estimated by Cusakin's equation:

9 $$k=\frac{0.732Q}{(2M-s)s}lg\frac{R}{r}, R=2s\sqrt{MK}$$

##### Calculation of the hydraulic fissure (pore) ratio

The relationship between fracture rate and groundwater flow quantity is as follows equation.

10 $$\mathrm{\varphi }=\frac{1}{577.9}\sqrt[3]{\frac{{{\text{K}}}_{{\text{c}}}\mathrm{\mu BlnR}/{\text{r}}}{{\text{H}}}}$$

1. $\mathrm{\varphi }$ is the fracture rate, %.
2. *B* is the volume coefficient of the liquid, m^3^/m^3^, *B* = 1.01.
3. *H* is the effective thickness of the aquifer, m. Unsealed aquifers in the well are considered as effective reservoirs.
4. $\mu$ is the viscosity of the water, CP(1CP = 1 mPa s).

The viscosity of water decreases with the gradual increase of temperature, and the change in viscosity will cause the flow rate to change exponentially (Table 5).

**Table 5:** The relationship between water temperature and viscosity.

Temperature T/℃	0	20	40	60	80	100
Viscosity $\upmu$/mPa s	1.792	1.005	0.656	0.469	0.357	0.234

According to the data in Table 5, the calculation equation of water temperature and viscosity is established by linear regression:

$$\mu =1.449{e}^{-0.019T}$$

In this equation, R^2^ = 0.9953. The groundwater temperature studied in this paper is in the range of 20–60 °C, and the error calculated by this equation can be controlled within − 3.3 ~ 1.2%.

(5) *k*_*c*_ is yield index. Using this equation:
  $${k}_{c}=\frac{Q}{\Delta p}$$
  where: *Q* is flow quantity, m^3^/d;$\Delta p$ is the pressure difference between dynamic and static water level, atmospheric pressure, bar (101,325 Pa).

##### Aquifer permeability coefficient calculated by actual flow velocity

According to Darcy's law, the relationship between aquifer permeability coefficient and groundwater velocity ($v$), pore or fissure rate ($n$), and hydraulic slope ($\frac{dh}{dl}$) are as follows:

11 $$k=nv\frac{dl}{dh}$$

Wher, $v$ the median value of the actual flow rate calculated by monitoring in the well is used to calculate the permeability coefficient, it is the ${k}_{1}$ in Table 6.

**Table 6:** Comparison of the permeability coefficient calculated by the two methods of flow velocity monitoring and pumping test.

Well number	Velocity $v$	Fissure ratio $n$	Hydraulic gradient $dh/dl$	Permeability coefficient ${\text{k}}$		Ratio ${k}_{1}:{k}_{2}$	Reliability
				Monitoring ${k}_{1}$	Pump test ${k}_{2}$		
	m/day	%	%	m/day	m/day		
DR01	16.14	0.06	3.93	0.26	0.25	1.04	Most credible
ZK106	21.45	0.29	8.56	0.73	0.08	9.16	Less credible
HT35	32.73	0.60	1.60	12.26	0.97	12.62	More credible
PH33	35.68	17.82	2.66	238.86	5.36	44.57	More credible
ZK01	27.67	0.31	0.62	13.80	0.08	172.5	Less credible

Reliability analysis of the calculated results. In November 2017, Well DR01 was completed and pumped. In July 2019, the well completed monitoring of groundwater flow direction. The author participated in these efforts. In June 2011, Well PH33 was completed and a pumping test was conducted. In December 2010, Well HT35 was completed, while pumping tests were conducted. In August 2019, the Well completed monitoring of groundwater flow direction. The author did not participate in well construction and pumping. No pumping test was carried out in ZK01 and ZK106 Wells, and the permeability coefficient and fracture rate of aquifer were referred to the pumping test results of nearby Wells (distance is 6.5 km and 21 km). Of the five Wells, DR01 had the most reliable data and the most reliable results. The results of HT35 and PH33 Wells are more reliable. Results from Wells ZK01 and ZK106 are less reliable and can only be used as reference.

As shown in the DR01 well, in the well, the hydrogeological parameters obtained by the pumping test and flow velocity monitoring method in the same time are the same. As shown in the Well PH33 and Well HT35, even in the same well, when the pumping test is separated by a long time of flow velocity monitoring (this is more than 8 and a half years apart), the hydrogeological parameters calculated by the two methods are large deviation. However, ZK01 and ZK106 show that when the pumping test and flow velocity monitoring are conducted in different Wells, the hydrogeological parameters obtained by the two methods are very different, even for the same aquifer. This may be caused by the difference of parameters of aquifers in different spatial locations.

#### Comparison of permeability coefficients

Be comparing with the permeability coefficient of aquifer calculated by the two methods (Table 6), the permeability coefficients of granite aquifer and schist aquifer the ${{\text{k}}}_{1}$ are close to the ${{\text{k}}}_{2}$, and the ratio is less than 10. Especially in the granite aquifers, ${{\text{k}}}_{1}$ and ${{\text{k}}}_{2}$ are nearly equivalent. In basalt aquifer and sand aquifer, there is a significant difference between the permeability coefficient k1 and the permeability coefficient k_2_, and the ratio is greater than 10. Especially in sand aquifers, and the difference is greater than 40 times. In Well ZK01, it is limestone aquifer, the permeability coefficient ${{\text{k}}}_{1}$ and ${{\text{k}}}_{2}$ varied greatly, and the ratio is more than 170 times. The permeability coefficient calculated by the actual velocity is closest to that obtained from the pumping test, in the same well and nearly time.

The reason for this gap is that, in the granite and schist aquifer, the flow of groundwater is controlled by the structural fissure surface, and the flow of water is more characterized by laminar flow. However, the basalt in well HT35 developed stomatal and almond-like structures, and the pore controls the flow of groundwater, and the water flow is both turbulent and laminar flow. In the sand layer, the pore is the space of groundwater movement, in which the groundwater flow is mainly for turbulent movement. In the limestone aquifer, the groundwater monitored this time is the karst cave and fissure water, and the water flow movement is more complex.

### Comparison between four different types of flow velocities in the same aquifer

We monitored the flow velocity of groundwater in five types of aquifers, including granite, schist, limestone, basalt and sand layer, and analyzed the differences between instantaneous flow velocity, time average flow velocity and cumulative flow velocity relative to the actual flow velocity in the four types of aquifers.

#### Granite aquifers

In granite aquifers, the time average velocity was 54% of its instantaneous velocity, its cumulative flow velocity was 46% of its time average velocity, and its actual flow velocity was 99% of its cumulative flow velocity. The groundwater in the structural fissures in granite aquifers exhibited four types of flow velocities between 186.82 and 635.17 μm/s, representing the largest difference of the four aquifer types (Table 7).The four types of velocities had the largest relative errors of 87%‒310%. Specifically, the instantaneous velocity had an error of + 238% relative to the cumulative flow velocity, the time average velocity had an error of + 81% relative to the cumulative flow velocity, the instantaneous velocity had an error of + 240% to the actual flow velocity, the time average velocity had an error of + 81% relative to the actual flow velocity, and the cumulative flow velocity had an error of + 1% relative to the actual flow velocity.

**Table 7:** Flow velocities of fissure water in basalts, limestones, schists and granites, and their ranking (unit: μm/s).

Lithology of aquifer		Schist	Limestone	Granite	Basalt	Sand
Instantaneous flow velocity	Value	423.63	504.31	635.17	415.42	453.90
	Rank	Fourth	Second	First	Fifth	Third
Time average velocity	Value	339.25	432.16	340.26	418.85	429.06
	Rank	Fifth	First	Fourth	Third	Second
Cumulative flow velocity	Value	292.19	304.78	188.30	348.72	412.92
	Rank	Fourth	Third	Fifth	Second	First
Actual flow velocity	Value	248.27	320.24	186.82	378.82	412.95
	Rank	Fourth	Third	Fifth	Second	First

#### Basalt aquifers

In Quaternary basalt aquifers, The time average velocity was 101% of theirs instantaneous velocity, theirs cumulative flow velocity was 83% of theirs time average velocity, and theirs actual flow velocity was 109% of theirs cumulative flow velocity. Moreover, theirs minimum cumulative flow velocity are 83% of its maximum instantaneous velocity, indicating only small differences between the four types of aquifers (Table 7). The four types of velocities were the closest to each other and had the smallest errors of -1% ~  + 10% relative to the actual flow velocity. Specifically, the instantaneous velocity had an error of + 16% relative to the cumulative velocity, the time average velocity had an error of + 17% relative to the cumulative flow velocity, the instantaneous velocity had an error of approximately + 9% relative to the actual flow velocity, the time average velocity had an error of + 10% relative to the actual flow velocity, and the cumulative flow velocity had an error of − 8% relative to the actual flow velocity.

#### Limestone aquifers

In limestone aquifers, the time average velocity was 86% of its instantaneous velocity, its cumulative flow velocity was 71% of its time average velocity, and its actual flow velocity was 105% of its cumulative flow velocity. These four types of flow velocities varied from 304.78 to 504.31 μm/s, and the cumulative flow velocity was 60% of the instantaneous velocity, indicating large differences (Table 7). The four types of velocities had small relative errors of − 5%‒58%. Specifically, the instantaneous velocity had an error of + 66% relative to the cumulative flow velocity, the time average velocity had an error of + 42% relative to the cumulative flow velocity, the instantaneous velocity had an error of + 58% relative to the actual flow velocity, the time average velocity had an error of + 35% relative to the actual flow velocity, and the cumulative flow velocity had an error of -5% relative to the actual flow velocity.

#### Schist aquifers

In schist aquifers, the time average velocity was 80% of its instantaneous velocity, its cumulative flow velocity was 86% of its time average velocity, and its actual flow velocity was 85% of its cumulative flow velocity. The metamorphic fissure water in schist aquifers exhibited four types of flow velocities between 248.54 and 423.63 μm/s; its actual flow velocity was 59% of its instantaneous velocity, indicating significant differences (Table 7). The four types of velocities had large relative errors of 18%‒71%. Specifically, the instantaneous velocity had an error of + 45% relative to the cumulative flow velocity, the time average velocity had an error of + 16% relative to the cumulative flow velocity, the instantaneous velocity had an error of + 71% relative to the actual flow velocity, the time average velocity had an error of + 37% relative to the actual flow velocity, and the cumulative flow velocity had an error of + 18% relative to the actual flow velocity.

#### Quaternary sand layer aquifer

In Quaternary sand layer aquifers, The time average velocity was 94% of theirs instantaneous velocity, theirs cumulative flow velocity was 96% of theirs time average velocity, and The actual flow velocity is almost equal to the cumulative flow velocity. Moreover, their minimum cumulative flow velocity are 91% of its maximum instantaneous velocity, indicating only small differences between the four types of flow velocity (Table 7). The four types of velocities were the closest to each other and had the smallest errors of 0 ~ 9% relative to the actual flow velocity. Specifically, the instantaneous velocity had an error of + 9% relative to the cumulative velocity, the time average velocity had an error of + 4% relative to the cumulative flow velocity, the instantaneous velocity had an error of approximately + 9% relative to the actual flow velocity, the time average velocity had an error of + 4% relative to the actual flow velocity, and the cumulative flow velocity had an error of -1% relative to the actual flow velocity.

From the above, the four types of groundwater flow velocities differed the most in granite aquifers. Specifically, they had a range of 154.83‒635.17 μm/s, and the instantaneous velocity was 4.10 times the cumulative flow velocity. They differed the least in Quaternary basalt aquifers. Specifically, they had ranges of 346.45‒416.70 μm/s and 412.92‒453.90 μm/s, with the instantaneous velocity 1.10–1.20 times the cumulative flow velocity. They had a range of 281.29–504.31 μm/s in limestone aquifers, with the instantaneous velocity 1.79 times the cumulative flow velocity, indicating a significant difference. In schist aquifers, they had a range of 274.80–423.63 μm/s, with the instantaneous velocity 1.54 times the cumulative flow velocity, indicating a small difference.

### The coefficient variation of the four types of flow velocity between different aquifers

In the four aquifers of schist, limestone, basalt and sand, the variation coefficient of groundwater velocity is that the variation coefficient of instantaneous velocity is greater than that of average velocity, and the variation coefficient of average velocity is greater than that of cumulative velocity. However, in granite aquifers, due to the different monitoring depth, the coefficient variation of average velocity is greater than that of cumulative velocity, and the coefficient variation of cumulative velocity is greater than that of instantaneous velocity (Table 8). In general, the coefficient variation of cumulative velocity is the smallest, which is more representative among the three velocity types. The fitting equation is established by using the "cumulative velocity–monitoring time" curve. The actual velocity is the limit value of the accumulated velocity when the time of the fitting equation tends to infinity. The actual velocity can further reduce the risk and is the most representative groundwater velocity.

**Table 8:** Coefficients variation for the different types of flow velocity.

Well number		ZK106	ZK01		DR01	HT35	PH33
Lithology of aquifer		Schist	Limestone		Granite	Basalt	Sand
Type of flow velocity	*V*_*i*_	0.57	0.36	0.43	0.35	0.39	0.49
	*V*_*t*_	0.37	0.28	0.31	0.48	0.23	0.24
	*V*_*c*_	0.1	0.19	0.23	0.41	0.06	0.18
	*V*_*a*_	–	0.04	0.18	0.47	0.09	0.19
Monitoring depth		Same monitoring depth		Different monitoring depth			

Monitoring the flow velocity obtained at the same depth in the same well, the coefficient variation of the four flow rates is shown as: the coefficient variation of the instantaneous velocity is greater than the time-averaged velocity, the coefficient variation of the time-averaged velocity is greater than that of the cumulative velocity, the coefficient variation of the actual velocity is smallest (Table 8). The fitting equation is established by using the "cumulative velocity–monitoring time" curve. The actual velocity is the limit value of the accumulated velocity when the time of the fitting equation tends to infinity. The actual velocity can further reduce the risk and is the most representative groundwater velocity.

The coefficient variation of actual velocity at different depths in the same well is studied. The coefficient variation depends on the uniformity of the storage space in the aquifer. In wells DR01, PH33, ZK01, and HT35, the coefficients variation of the actual velocity were 0.47, 0.19, 0.18, and 0.09, respectively. The coefficient variation of the actual velocity is the largest in the DR01 well, and the fissure water velocity varies greatly at different depths, because the fissure water content space in the granite is uneven. The coefficient variation of actual velocity in well HT35 is the smallest, the difference in the flow velocity at different depths is small, and the hole crack in the basalt aquifer is relatively uniform (Table 8).

## Conclusions

1. The colloidal borescope, combined with in-well cameras, deep-well devices for stabilization and catch, and a high-resolution monitoring system based on layered hydraulic isolation, contributed to the precise positioning of the target fissures and aquifers to be monitored, the control of probe swinging in a well during data acquisition, and the shielding of the interference in the flow velocities and directions caused by the vertical flow of groundwater in the well. The colloidal borescope therefore allows the acquisition of high-precision data. The magnitude and directions (azimuths) of instantaneous velocity obtained via monitoring using the colloidal borescope were converted into N–S and W–E components in the rectangular coordinate system, and the N–S and W–E components of the time average velocity were calculated according to selected time intervals. This process reduced the data redundancy caused by the excessive data and uneven distribution of the instantaneous velocity.
2. The actual flow velocity of groundwater can be calculated by limit analysis of the cumulative flow velocity *vs.* monitoring time curve-fitting equations. Multiplying the N–S and W–E components of the time average velocity of a certain time interval with the time interval yields the N–S and W–E displacement of the particles, respectively. The displacement trajectory of particles can be plotted using the accumulated displacement of multiple time intervals. The cumulative displacement and azimuth of the particles at a certain time can be calculated using the N–S and W–E displacement components of the particles relative to the origin (starting point for calculation) of various time intervals. The ratio of the cumulative displacement in a certain time to the time is referred to as the cumulative flow velocity. The cumulative flow velocity consisted of a set of continuous data from the beginning to the end of the calculation and was used to plot the cumulative flow velocity *vs*. monitoring time curves. The curves were divided into L, Γ, V^−^, and Λ___ shaped curves, and their curve-fitting equations were obtained using exponential or logarithmic equation models. The cumulative flow velocity of groundwater over a longer monitoring time was closer to its actual flow velocity at the monitoring sites. The actual flow velocity was taken to be the limit of the cumulative flow velocity of groundwater as the monitoring time tends to infinity and was calculated using the cumulative flow velocity *vs.* monitoring time curve-fitting equations.
3. To improve work efficiency, different methods were selected to monitor and calculate the flow velocities and directions of fissure water in different aquifers, according to different precision requirements. The most effective method for obtaining high-precision flow velocities and directions of fissure water in bedrock is the actual flow velocity method. The flow velocities and directions of metamorphic-rock fissure water and the fissure water in caves with errors of < 5% can only be obtained using this method. The cumulative flow velocity method is widely applicable to the four types of fissure water in bedrock when the error requirement is < 20%, and the time average velocity method is applicable to the four types of fissure water in bedrock when the error requirement is < 50% (Table 9). However, it is not recommended that the instantaneous velocity method be used to monitor and calculate the flow velocities and directions of the fissure water in granites owing to the significant errors that arise.
4. The instantaneous velocity, time average velocity, and cumulative flow velocity of groundwater in different aquifers have different errors relative to the actual flow velocity. The three types of flow velocities of the fissure water in the vesicles Quaternary were the closest to each other and had the smallest errors of − 1%‒11% relative to the actual flow velocity. Specifically, the instantaneous velocity had an error of + 10%‒+ 20% relative to the cumulative velocity, the time average velocity had an error of + 4%‒+ 20% relative to the cumulative flow velocity, the instantaneous velocity had an error of approximately + 10% relative to the actual flow velocity, the time average velocity had an error of + 4%‒+ 11% relative to the actual flow velocity, and the cumulative flow velocity had an error of − 8%‒ + 0% relative to the actual flow velocity. The three types of flow velocities of the fissure water in solution-enlarged fractures of limestones had a small relative error of − 5%‒58%. Specifically, the instantaneous velocity had an error of + 66% relative to the cumulative flow velocity, the time average velocity had an error of + 42% relative to the cumulative flow velocity, the instantaneous velocity had an error of + 58% relative to the actual flow velocity, the time average velocity had an error of + 35% relative to the actual flow velocity, and the cumulative flow velocity had an error of − 5% relative to the actual flow velocity. The three types of velocities of fissure water of schist had a large relative error of 18%‒71%. Specifically, the instantaneous velocity had an error of + 45% relative to the cumulative flow velocity, the time average velocity had an error of + 16% relative to the cumulative flow velocity, the instantaneous velocity had an error of + 71% relative to the actual flow velocity, the time average velocity had an error of + 37% relative to the actual flow velocity, and the cumulative flow velocity had an error of + 18% relative to the actual flow velocity. The three types of velocities of fissure water of granites had the largest relative error of 87%‒310%. Specifically, the instantaneous velocity had an error of + 238% relative to the cumulative flow velocity, the time average velocity had an error of + 81% relative to the cumulative flow velocity, the instantaneous velocity had an error of + 240% to the actual flow velocity, the time average velocity had an error of + 81% relative to the actual flow velocity, and the cumulative flow velocity had an error of + 1% relative to the actual flow velocity.

**Table 9:** Suggestions on error control and monitoring methods.

Groundwater type	Error control target (%)				
	≦ 5	5 ~ 10	10 ~ 20	20 ~ 50	50 ~ 100
Metamorphic fissure water of schists	*V*_*a*_	*V*_*a*_	*V*_*c*_	*V*_*t*_	*V*_*i*_
Tectonic fissure water of granites	*V*_*c*_	*V*_*c*_	*V*_*c*_	*V*_*c*_	*V*_*t*_
Karst fissure water of limestones	*V*_*c*_	*V*_*c*_	*V*_*c*_	*V*_*t*_	*V*_*i*_
Fissure water in basalt holes	*V*_*a*_	*V*_*c*_	*V*_*i*_, *V*_*t*_, *V*_*c*_		
Loose sand pore water	*V*_*c*_	*V*_*t*_	*V*_*i*_, *V*_*t*_		

*V*_*i*_ instantaneous flow velocity method, *V*_*t*_ time average velocity method, *V*_*c*_ cumulative flow velocity method,* V*_*a*_ actual flow velocity method.

## Data Availability

The datasets used and/or analyzed during the current study are available from the corresponding author on request.
